# Detection of the amoeba *Entamoeba gingivalis* in periodontal pockets

**DOI:** 10.1051/parasite/2014029

**Published:** 2014-07-02

**Authors:** Mark Bonner, Véronique Amard, Charlotte Bar-Pinatel, Frédéric Charpentier, Jean-Michel Chatard, Yvan Desmuyck, Serge Ihler, Jean-Pierre Rochet, Véronique Roux de La Tribouille, Luc Saladin, Marion Verdy, Núria Gironès, Manuel Fresno, Julien Santi-Rocca

**Affiliations:** 1 Association Médicale contre les Infections Buccales (AMIB) Nice France; 2 Institut International de Parodontie Nice France; 3 Immune Activation Laboratory, Centro de Biología Molecular Severo Ochoa, Consejo Superior de Investigaciones Científicas, Universidad Autónoma de Madrid, Cantoblanco Madrid Spain

**Keywords:** *Entamoeba gingivalis*, Periodontitis, PCR, Microscopy, Diagnosis, Protist, Amoeba

## Abstract

Periodontitis is a public health issue, being one of the most prevalent diseases worldwide. However, the aetiology of the disease is still unclear: genetics of patients cannot explain the dispersed or isolated localisation of gingival pockets, while bacteria-based models are insufficient to distinguish gingivitis and periodontitis. The possible role of parasites in the establishment of periodontitis has been poorly studied until now. The aim of this project was to study a potential link between colonisation of gingival crevices by the amoeba *Entamoeba gingivalis* and periodontitis. In eight different dental clinics in France, samples were taken in periodontal pockets (72) or healthy sites (33), and submitted to microscopic observation and molecular identification by PCR with a new set of primers designed to specifically detect *E. gingivalis*. This blind sample analysis showed the strong sensitivity of PCR compared with clinical diagnosis (58/72 = 81%), and microscopy (51/65 = 78%). The results of this work show that the parasites detected by microscopy mainly – if not exclusively – belong to the species *E. gingivalis* and that the presence of the parasite is correlated with periodontitis.

## Abstract (fr)

La parodontite est un problème de santé publique, car c’est l’une des maladies avec la plus forte prévalence au monde. Cependant, son étiologie est à ce jour incertaine : la génétique des patients ne peut pas expliquer la localisation dispersée ou isolée des poches parodontales, alors que les modèles basés exclusivement sur la colonisation bactérienne sont insuffisants pour distinguer gingivite de parodontite. Le rôle éventuel des parasites dans l’établissement de la parodontite a été peu étudié jusqu’à présent. Le but de ce projet était d’étudier le possible lien entre la parodontite et la colonisation des sillons gingivaux par l’amibe *Entamoeba gingivalis*. Dans huit cabinets de parodontologie en France, des échantillons pris dans des poches parodontales (72) ou des sillons sains (33) ont été soumis à observation microscopique et identification moléculaire par PCR grâce à une nouvelle paire d’amorces dessinées pour détecter spécifiquement *E. gingivalis*. Cette étude en aveugle a montré une forte sensibilité de la PCR par rapport à la clinique (58/72 = 81 %) et à la microscopie (51/65 = 78 %). Les résultats de ce travail montrent que les parasites détectés par microscopie appartiennent majoritairement – sinon exclusivement – à l’espèce *E. gingivalis* et que la présence du parasite est corrélée à la parodontite.

## Introduction

Periodontitis is one of the most prevalent diseases worldwide. The pathology is characterised by gum inflammation with bone loss, often associated with pain, halitosis and gingival bleeding. In frequent cases, alveolysis can lead to tooth loosening or even loss. Though these clinical manifestations are obvious signs of disease, patients habitually tolerate discomfort or suffering; the impact of oral health-related quality of life is indeed often underestimated [20].

Health professionals face a challenging issue with periodontitis: unlike most diseases, the aetiology of periodontitis is not clearly determined. Thus, rational design of therapeutic care is impossible and based on imperfect modelisation. Indeed, the corpus of literature available on periodontitis mainly focuses on a possible bacterial aetiology, aggravated by genetic determinants of the patients. However, this hypothesis fails to explain the difference between gingivitis and periodontitis. Animals have been used to develop models for the study of periodontitis [17]. Surprisingly, parasites were not sought in the studies on these different models; their role in the pathology cannot be ruled out, especially in human, non-experimental periodontitis.

As early as 1849, amoebae were detected in the mouth by G. Gros: “*Endamoeba gingivalis*” was the first symbiotic amoeba described in humans [8]. Though it was observed in patients suffering periodontitis, the putative aetiological link between the later-called “*Entamoeba gingivalis*” parasite and periodontal disease was left neglected for more than a century.

In the early 1980s, new interest in *E. gingivalis* arose after T. Lyons detected amoeboid organisms in periodontal pockets, while they were absent from healthy sites [14]. Assuming these parasites were responsible for periodontal disease, he implemented a new therapeutic protocol, including oxygen peroxide and metronidazole [15]. This was effective, as confirmed by a recent study [4].

These assumptions were contrasted by cautiousness from the scientific and medical communities, who encouraged molecular identification of the parasite. Two main studies have already been published, leading to disparate results, from roughly 6% to 69% prevalence of *E. gingivalis* in periodontal pockets [11, 23]. We propose to give new insight into this controversial issue. As microscopic diagnosis cannot determine the species of the detected amoeboid organism, we designed new molecular tools and experimental procedures to identify *E. gingivalis* unequivocally in crevicular material in a multi-site epidemiological study.

## Material and methods

### Ethics statement

Written consent was obtained from all patients, informing them that samples would only be dental plaque leftovers from normal consultations. No additional sampling would be needed for the study: these leftovers would normally be discarded. The samples were used anonymously and patients never received any feedback from the treatment of their samples, nor about the possible unsuitability of the sample (e.g. volume, exclusion for previous treatment) to be included in the study. Patients were also informed that samples would not be used for commercial/industrial purposes (research and non-profit aims). This was in compliance with local laws and the corresponding authority of the Centro de Biología Molecular Severo Ochoa (Madrid, Spain).

### Patient cohort

One hundred and thirty-nine patients consulting in eight dental clinics in France (34 in Troyes, 27 in Château-Thierry, 20 in Nantes, 20 in Le Blanc-Mesnil, 15 in Rozay-en-Brie, 12 in Bonneville, 9 in Lyon and 2 in Saint-Maur-des-Fossés) were proposed for this study. Recruitment took place between May and December 2011. Exclusion parameters were: antibiotic treatment in the last few months, chronic diseases or suspicion of pregnancy. In cases of a positive answer to one or several of these questions, samples were taken but rejected without informing the patient. Sex, age and socio-economic or behavioural characteristics (smoking, hygiene) were recorded only in the practitioners’ files. Thus, the classical visit for the patient was not modified, except for signing the informed consent form.

### Clinical diagnosis of periodontitis

During the visit, practitioners trained by the AMIB association and in accordance with the main periodontology and parasitology researchers involved in the study determined the presence of periodontitis sites and reported on a form the sample number, the affected tooth, the dental face, the presence of oedema (+ or −), bleeding (+ or −), the pocket’s depth (depth ≥ 3 mm was considered positive), gum recession (recession ≥ 1 mm was considered positive) and mobility (index from 0 to 3, ≥2 was considered positive). If two or more parameters were positive, the corresponding sulcus was considered to be affected by periodontitis, according to clinical parameters. The results were recorded in a database that was not accessible to the molecular biologist performing PCR experiments.

### Microscopic diagnosis

Only one site was used for each patient. In periodontitis or healthy sites, periodontal material including dental plaque was sampled with a probe. The sample was saliva-mounted and immediately observed by phase-contrast microscopy. When a sufficient amount of plaque was sampled, the leftover was kept for PCR and thus included in the study. The patient was not informed when the sample could not be used for PCR. Amoebae were detected by their producing one lobose pseudopodium at a time, with dark intracellular vacuoles and one nucleus containing a central karyosome and peripheral chromatin (Supplemental Fig. S1). Amoeboid movement is easy to detect, as well as phagocytic activity. In case of doubt – for instance, if the nucleus could not be clearly detected – the cell was not recorded as an amoeba. A positive sample corresponded to a sample in which at least one amoeba was detected. The results were recorded in a database that was not accessible to the molecular biologist performing PCR experiments.

### Sample preparation for PCR

The part of the sample dedicated to PCR was plunged into 50 μL medium we called TEGI (100 mM Tris pH 8.0; 10 mM EDTA; 5 M guanidine isothiocyanate). This medium allows the lysis of the sample and its conservation at room temperature. Kept away from light, samples were shipped without any indications but the sample number. Upon arrival, samples were diluted with 450 μL water and 4 units of proteinase K (5 μl; P4850, Sigma) were added before overnight incubation at 56 °C with shaking. A double extraction was performed with 500 μL phenol-chloroform-isoamyl alcohol 25:24:1 saturated with 10 mM Tris, pH 8.0, 1 mM EDTA. The aqueous phase was subsequently extracted with 500 μL chloroform and precipitated with 50 μL sodium acetate 3 M, pH 5.2, and 900 μL pure ethanol. Rinsed two times with 70% ethanol, samples were dried and resuspended with 50 μL nuclease-free water. Purity was assessed by spectrophotometry (NanoDrop 1000, Thermo Scientific) using absorbances at 230, 260, 270, 280 and 340 nm. When validated, samples were diluted at 10 ng/μL (unless stated).

### PCR

**Figure 1.**
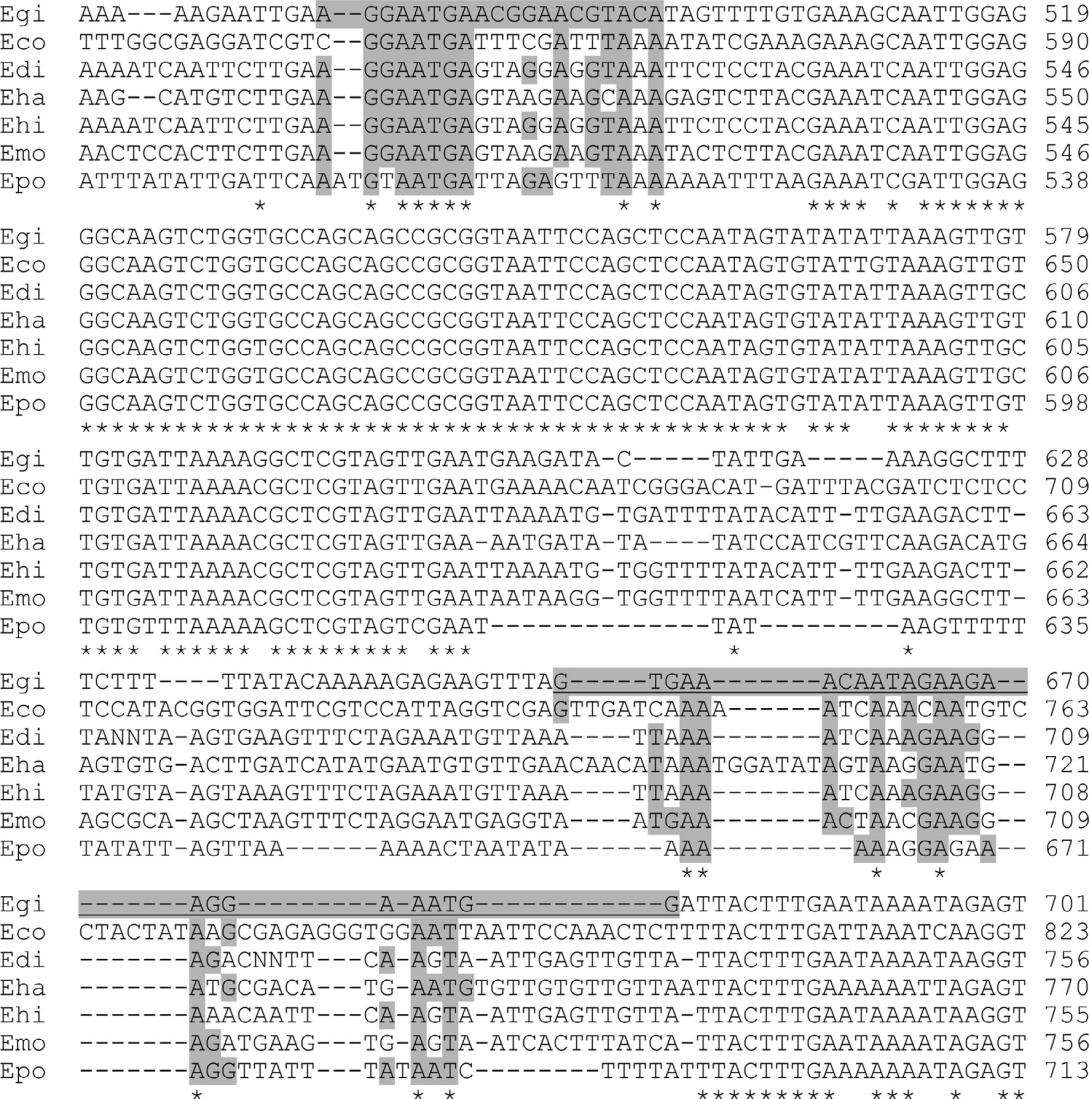
Small subunit ribosomal RNA sequence comparison for *Entamoeba* species found in humans. *E. gingivalis* (Egi), *E. coli* (Eco), *E. dispar* (Edi), *E. hartmanni* (Eha), *E. histolytica* (Ehi), *E. moshkovskii* (Emo) and *E. polecki* (Epo) sequences are displayed. The forward primer used in this study is shaded, the reverse complement sequence to reverse primer is shaded and underlined.

For *E. gingivalis* diagnosis, reaction was performed in 25 μL, with 1U Taq polymerase (FastStart Taq DNA polymerase, Roche), 2.5 mM MgCl_2_, 200 nM of each dNTP and 1 μM of each primer. The primers we designed are presented in the context of the SSU rDNA gene in Figure 1 and Supplemental Figure S2 (sequences: 5′-AGGAATGAACGGAACGTACA-3′ and 5′-CCATTTCCTTCTTCTATTGTTTCAC-3′). DNA matrix was used at 40 ng per reaction, unless stated. Spiking was performed with 100 pg patient #0 DNA. Primers against a 151-bp human sequence (Accession number NT_032977.9) were a kind gift of Dr. Germán Andrés Hernández (CBMSO, Madrid) and were used in similar conditions (sequences: 5′-CAATGCCTCCTGCACCAC-3′, 5′-CCATCACGCCACAGTTTCC-3′). The PCR programme was as follows: initial denaturation (94 °C, 3′30ʺ), 40 cycles (94 °C, 1′; 60 °C, 1′; 72 °C, 1′), final extension (72 °C, 1′) and conservation (4 °C). PCR for other *Entamoeba* species was performed as described elsewhere [10]. Samples (10 μL) were resolved on 2% agarose gel with suitable DNA molecular weight markers (Thermo Scientific SM0241).

### Data analysis

Once all experiments were performed and the final results definitively recorded, data from the eight clinics using the three methods were merged and analysed. Statistical analysis for association was done using Pearson’s chi-squared test.

## Results

### Genetic differences in the *Entamoeba* genus

The *Entamoeba* genus has been the subject of numerous genetic studies, in particular to identify virulence factors of the pathogenic organism *Entamoeba histolytica*. In several studies, new amoebae resembling *E. histolytica,* identified in samples from animals, allowed the creation of phylogenetic trees of the *Entamoeba* genus according to their small subunit ribosomal DNA sequence [21]. Amoebae able to colonise the human host are spread within the genus, according to the sequence of this gene; however, reflecting their differences in nucleus number of their cyst form. The mouth-colonising *E. gingivalis* has no identified cyst form, despite its vicinity to species producing mono- or tetranucleated cysts, according to their small subunit ribosomal DNA sequences. We sought regions in this sequence that would allow differential molecular diagnosis of *E. gingivalis*. Sequences from all species colonising humans were aligned (Fig. 1) and two PCR primers were designed in regions diverging in *E. gingivalis*. It is noteworthy that no cross-reactivity was detected for any genomic sequences for any species (including bacteria) available in the BLAST database (http://www.ncbi.nlm.nih.gov/Blast.cgi), including all the other *Entamoeba* species. Primers from previous studies and the whole gene sequence are shown in Supplemental Figure S2.

### Development of a sensitive, species-specific PCR for *E. gingivalis*

**Figure 2.**
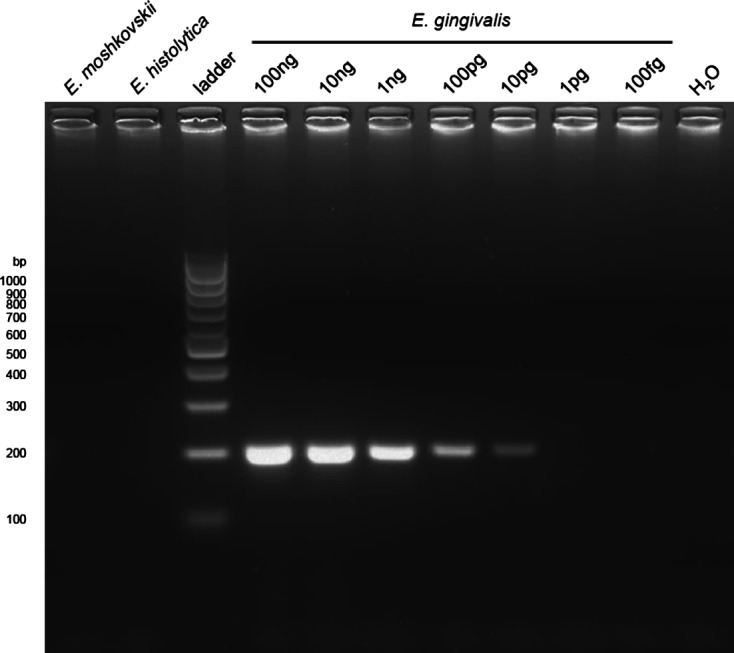
Specificity and sensitivity of *Entamoeba gingivalis*-specific primers. Agarose gel-resolved products from single-round PCR using 1 ng *E. moshkovskii*, 1 ng *E. histolytica* and 100 fg–100 ng *E. gingivalis* patient #0 DNA. Amplification was not obtained in control without matrix (H_2_O), *E. moshkovskii*, *E. histolytica* and up to 10 pg patient #0 DNA.

The specificity of previous primers was assessed using DNA from two closely-related species: *Entamoeba moshkovskii* and *E. histolytica*. No amplification was detected with 10 ng DNA from these matrices, nor with water controls (Fig. 2). Purulent dental plaque containing motile amoebae from a patient with aggressive periodontitis (patient #0) was used as a positive control. Special attention was drawn to DNA purification, achieved by cell lysis and a double phenol-chloroform extraction, followed by a chloroform extraction and a sodium acetate precipitation with two wash steps. Spectrophotometer analysis of the sample revealed the high purity of the DNA sample (DNA sample #0). This sample contained a high number of bacteria, amoebae and human leucocytes. So, the number of amoebae for a given amount of DNA for sample #0 is lower as compared with pure amoeba DNA from cultured trophozoites, as was the case for *E. moshkovskii* and *E. histolytica*. However, amplification of a DNA fragment of the expected (203 bp) size was detected for amounts as low as 10 pg DNA from patient #0. An *E. histolytica* trophozoite contains approximately 40 fg, a bacterium, around 4 fg, and a human cell roughly 6 pg DNA. In conclusion, the high sensitivity of the method and its specificity for *E. gingivalis* allowed us to confirm that exclusive detection of this parasite was possible by a single-round PCR assay.

### *Entamoeba gingivalis* is distinct from other species

**Figure 3.**
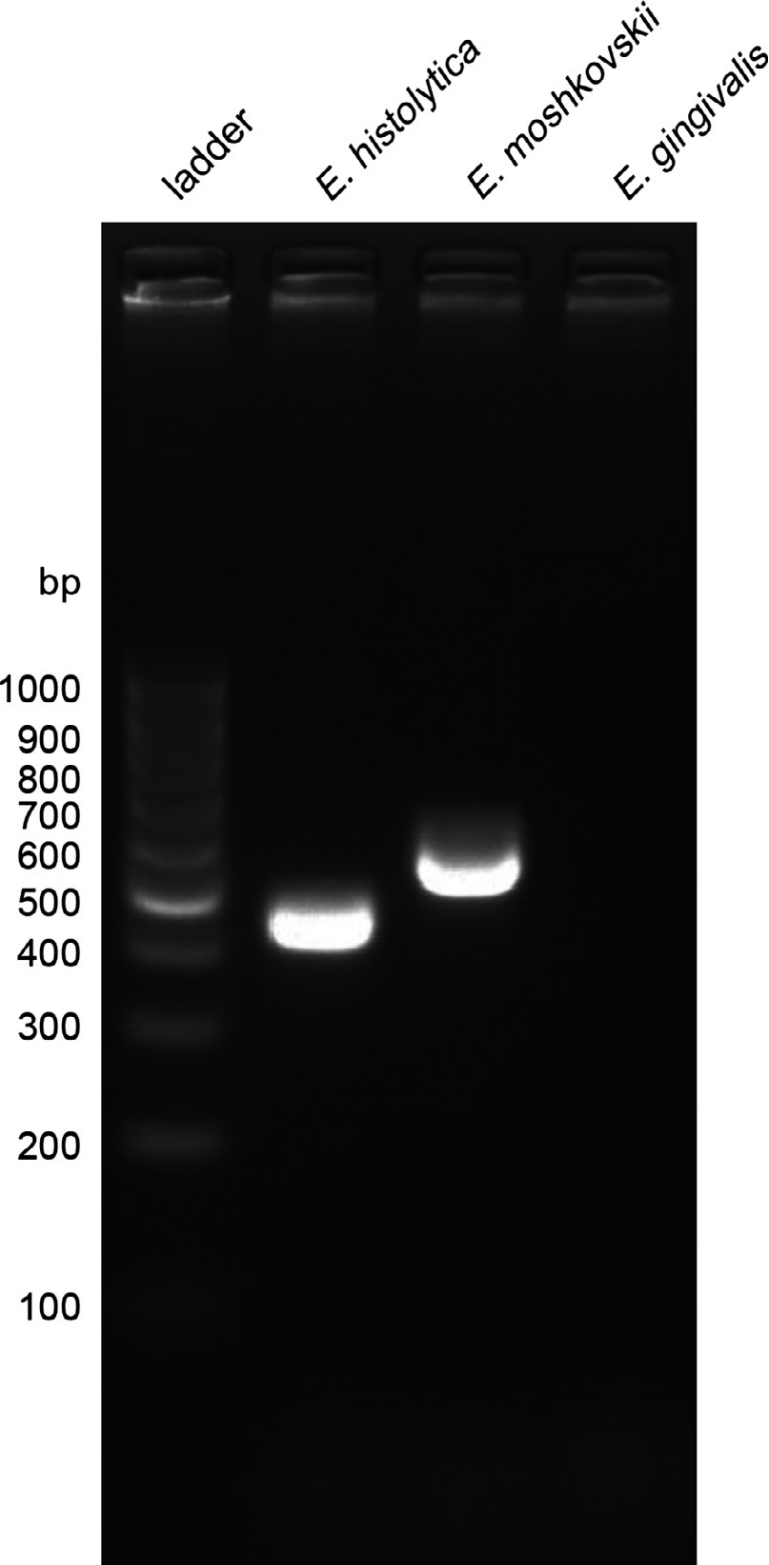
*Entamoeba gingivalis* is not detected by primers specific for other species of the *Entamoeba* genus. Agarose gel-resolved products from nested PCR using 1 ng *E. moshkovskii*, 1 ng *E. histolytica* and 125 ng *E. gingivalis* patient #0 DNA. Amplification was not obtained in control without matrix (not presented) and *E. gingivalis*.

In the parasite *E. histolytica*, ribosomal genes are organised in arrays repeated in episomal circles [3]. In other parasites, hybrid genetic lineages are observed, as in the kinetoplastid parasite *Trypanosoma cruzi* [22]. In the absence of extensive studies, the potential existence of hybrids or subspecies cannot be ruled out. Thus, the presence of various types of circles offering different rDNA sequences is not yet excluded and could cause a repositioning of the species *E. gingivalis* in the phylogenetic tree of the genus *Entamoeba*. To avoid this type of confusion, we used primers with sequences specific to *E. moshkovskii*, *Entamoeba dispar* and *E. histolytica* for a high-sensitivity, nested PCR assay [10]. This method allowed detection of *E. moshkovskii* and *E. histolytica* with 1 ng DNA matrix, with amplicons at specific, expected sizes (553 and 439 bp, respectively; Fig. 3). However, with 100 ng DNA from patient #0 (*E. gingivalis*), no amplification was detected. This was a supplemental clue that the parasites detected by the PCR we designed belong to a species distinct from *E. moshkovskii*, *E. dispar* and *E. histolytica*, corresponding to a previously described sequence associated with the parasite *E. gingivalis* [11]. We could thus conclude that our method was a *bona fide* diagnosis for the parasite *E. gingivalis*.

### Controlling negative PCR results

**Figure 4.**
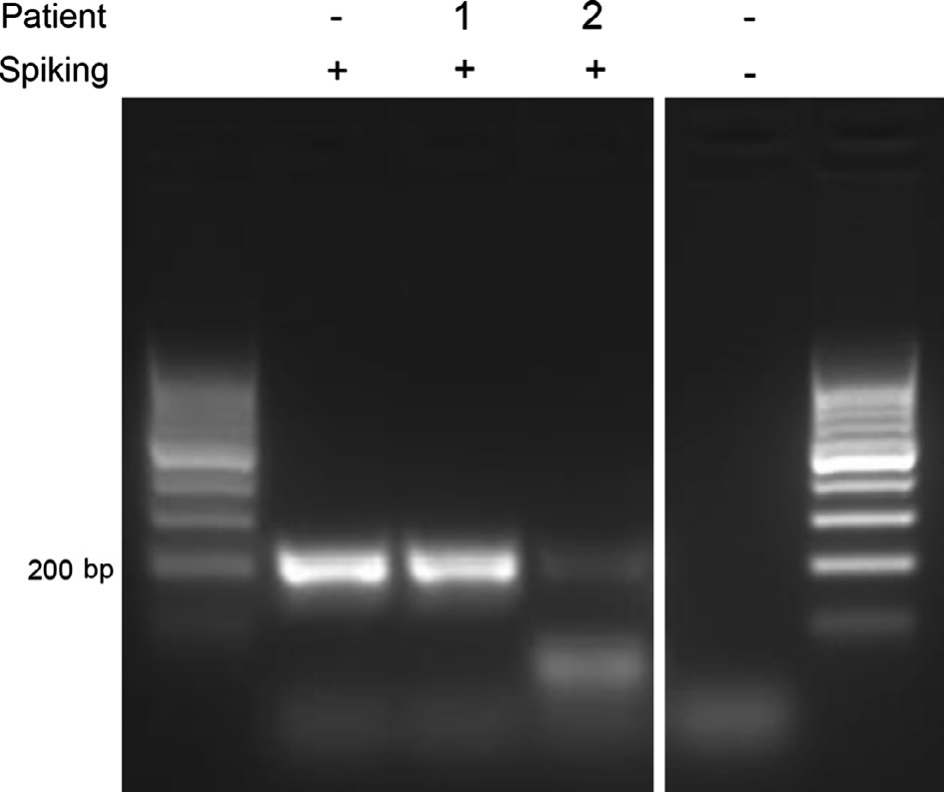
Detection of inhibitors in matrices. PCR reactions for *Entamoeba gingivalis* detection with 40 pg DNA matrix from patients 1 or 2 were spiked with 100 pg DNA matrix from patient #0. For patient 2’s sample (fourth lane), a fainter band of 200 bp is observed in comparison with the second lane (spiking only) or third lane (uninhibited patient 1 sample), revealing the presence of inhibitors in this sample. A control without matrix or spiking was included (fifth lane).

Negative results from PCR can result from the absence of the DNA target in the sample, its degradation, or the inhibition of its amplification. Inhibition can be due to the presence of salts (e.g. magnesium salts), chemical products (e.g. phenol), inhibitors of enzymes (e.g. antibodies as used for hot start, or haemoglobin), or molecules modifying or chelating primers (e.g. complementary RNA forming more stable hybrids). All cases except the last one can be controlled by amplifying another gene in the sample, for instance a bacterial gene, as was done in previous studies [23]. However, gene-specific inhibitors cannot be controlled this way and can be studied by spiking negative matrices with limiting amounts of positive DNA matrix from patient #0 (Fig. 4). Limiting amounts were defined in a first experiment as the last tenfold dilution of total DNA producing 100% positive amplifications (assayed with 10 amplifications giving unambiguous bands): we determined it corresponded to 100 pg of total DNA. In the absence of inhibitors in the negative matrix, the sequence from the #0 matrix was amplified (patient 1 in Fig. 4). Otherwise, amplification of #0 DNA was inhibited: no detection was possible or a fainter band was observed (patient 2 in Fig. 4). This method allowed us to reject samples that would have been considered as negative, though we cannot conclude about their initially containing target DNA from *E. gingivalis*.

During purification, DNA could possibly not be retrieved – for instance, if the nuclear membrane was not efficiently lysed during the first steps – or could be degraded. We chose to amplify a single-copy, nuclear sequence (the human gene, with no homology with any *Entamoeba* sequences) from human cells of the same size as the amoebic target. In all negative samples without inhibition, this sequence was detected, indicating that DNA degradation did not impede its amplification. We thus concluded that it was improbable that the lack of detection of *E. gingivalis* target DNA was due to DNA degradation.

### Epidemiological study

**Figure 5.**
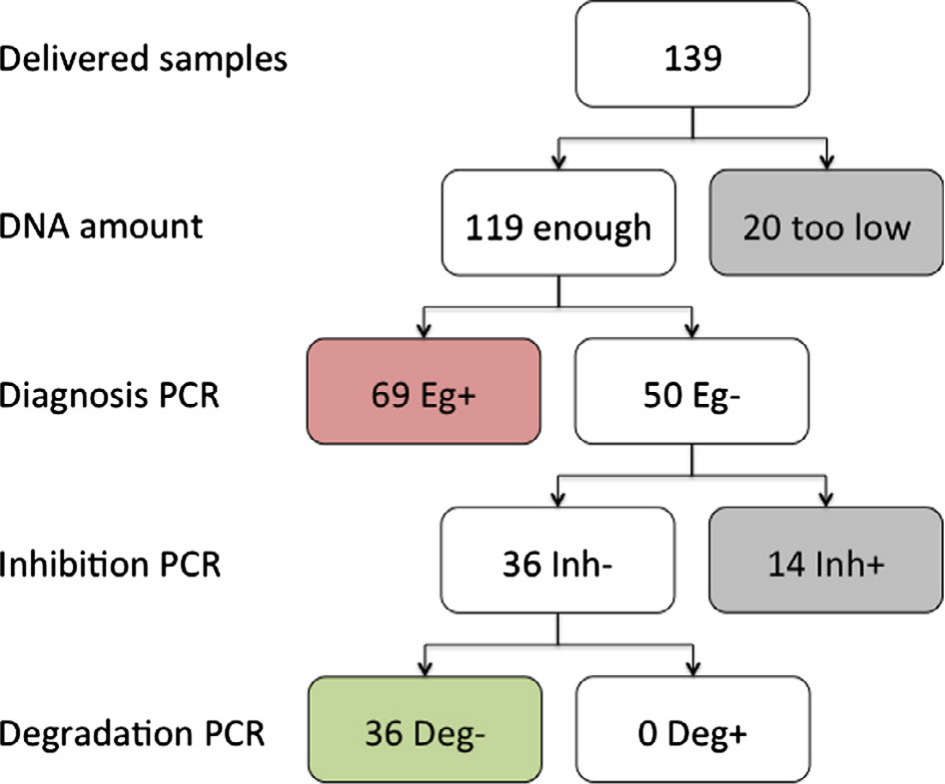
Sequential diagram of sample treatment. Numbers of samples at different steps of their treatment are presented, in particular results from the detection of *Entamoeba gingivalis* by PCR (“Eg+” stands for positive results; “Eg−”, for negative results), the presence or absence of inhibitors (“Inh−” and “Inh+”, respectively), and degradation or not of DNA (“Deg+” and “Deg−”, respectively).

From the 139 samples received, 85.6% (119/139) gave sufficient amounts of DNA to proceed to the PCRs. After the first PCR, 60% (69/119) of samples allowed amplification of a 203-bp fragment, as expected for *E. gingivalis* diagnosis. Thus, 50 samples (42%) were negative (Fig. 5). The presence of inhibitors was detected in 28% (14/50) of these samples. In the 72% (36/50) negative samples without inhibitors, detection of a 151-bp human sequence was achieved, indicating that lack of *E. gingivalis* SSU rDNA target sequence amplification was unlikely to be due to DNA degradation. Therefore, after all controls, 105 samples were considered exploitable: 69 positive and 36 negative.

Blinded databases were then gathered and results were compared among the three methods (Table 1). By clinical observation, 68.6% (72/105) of samples were considered positive and 31.4% (33/105) negative; this method being considered the “gold standard”, we thus concluded that we used a cohort of patients with a prevalence for periodontitis of 68.6% (72/105). It is noteworthy that the majority (70%) of samples from which we could not purify enough DNA were clinically negative (14 periodontitis negatives by clinical observation from 20 samples with low/no purified DNA). Interestingly, inhibited samples were mainly (78.6%) from patients without periodontitis (11 periodontitis negatives by clinical observation from 14 inhibited samples). These results and the blind treatment of the samples explain the lower number of healthy donors included in the study.

**Table 1.** Comparison of diagnosis methods and statistical indicators. PCR was compared with clinical diagnosis (a), and with results obtained by microscopy (b); diagnosis by microscopy was also compared with clinical diagnosis (c).

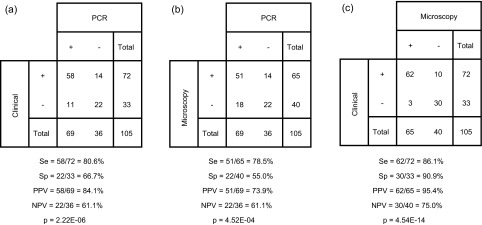

Microscopic detection of *E. gingivalis* and clinical diagnosis of periodontitis are often considered synonymous. Indeed, the discovery of this parasite in periodontal pockets [8] and pioneer explorations only with periodontitis patients that were all positive by microscopy [13] can be misleading. In the present study, statistical indicators for microscopy in comparison with clinical diagnosis were very high (sensitivity = 86.1% (62/72), specificity = 90.9% (30/33), positive predictive value = 95.4% (62/65), and negative predictive value = 75% (30/40), association (Pearson’s chi-squared test): *p* = 4.54 × 10^−14^). Importantly, this is the first study in which healthy patients were recruited: in 90.9% of them (30/33), amoebae were not detected by microscopy.

PCR diagnosis showed lower statistical indicators as compared with the other methods, but still associated with them (*p* = 2.22 × 10^−6^ and *p* = 4.52 × 10^4^ as compared with clinical and microscopic diagnoses, respectively). Interestingly, the sensitivity was still high (80.6% (58/72) and 78.5% (51/65), as compared with clinical and microscopic diagnoses, respectively) while the specificity was much lower (66.7% (22/33) and 55% (22/40)). This means that the PCR allowed the detection of *E. gingivalis* DNA in samples that were from patients without damage (33.3% = 11/33) or without amoebae visible by microscopy (45% = 18/40).

**Figure 6.**
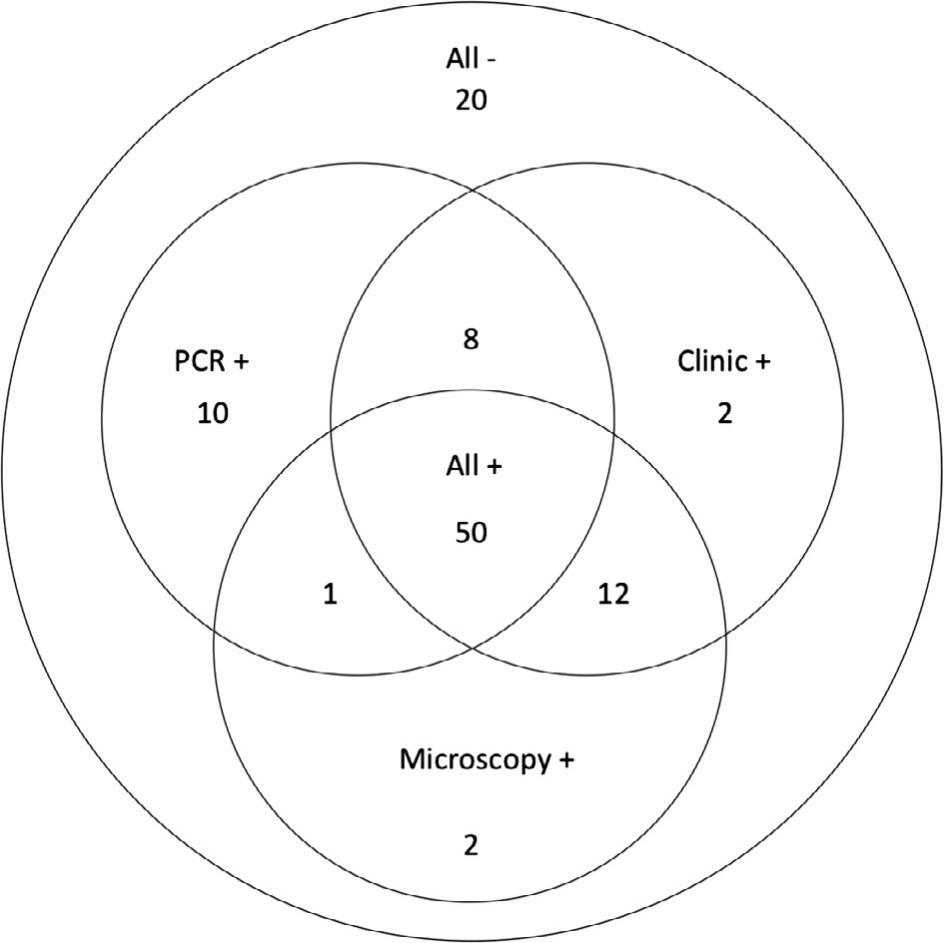
Venn diagram of results. Comparison of the results for the three diagnostic methods.

To represent correlations and divergences between methods better, we summed up the results in a Venn diagram (Fig. 6). This informative approach permitted us to determine that 50 positive and 20 negative samples were concordant for the three methods (66.7% of concordant results (70/105)). However, noticeable divergences were highlighted by this analysis: 12 samples were negative and 10 positive only for PCR. This result may be due to the fact that, beyond differences in the sensitivity and specificity of the methods, these methods are based on different diagnostic targets: genetic (PCR), morphological (microscopy), and clinical parameters. These detection methods may not be synonymous and their results may have different biological and pathophysiological meanings, as will be discussed further.

## Discussion

The gastrointestinal tract is at the interface between the human body and the external milieu. The external layer of this system is composed of various epithelia, which constitute barriers guaranteeing the integrity of the organism and protecting it from invasion by foreign microbes. Bacteria in the gastrointestinal tract can be beneficial: for instance, ruminants digest cellulose thanks to their microbiota. In humans, the concept of commensal microbiota is generally accepted; however, its beneficial effect is often misunderstood. The beneficial microbiota of the upper counterpart of the tract is less documented, though some concepts are shared: some bacteria are associated with lesser prevalence of some pathologies, such as *Porphyromonas catoniae* and *Neisseria flavescens* [6], though their taking part in the protective process against cavities cannot be claimed without further investigation. These bacteria could only be passive indicators of oral health.

Such is the status of many pathology-associated bacteria, for which the aetiological involvement in disease has never been proven. In the case of *E. gingivalis*, the causative link between the presence of the parasite and development of periodontitis has never been demonstrated. However, in our study and precedent work, it has been shown that *E. gingivalis* is infrequently detected in healthy donors [11, 23]. Providentially, the concept of incubation for infectious diseases is commonly known and the detection of *E. gingivalis* prior to the onset of periodontitis does not impede us from speculating that it can be the causative agent of the disease. Spontaneous control and healing could also occur in patients, depending on their genetic background, their health status, and possible genetic differences in the parasites, as observed in *E. histolytica* [1, 2]. Thus, the apparently discordant results we observed in particular patients negative for clinic and positive for PCR could be explained by a better understanding of the interactions between *E. gingivalis* and its mammalian host.

Interestingly, the 12 patients in this study that are negative for PCR and positive for both clinic and microscopy could be explained by different phenomena. First, we could hypothesise an unfortunate heterogeneity of the samples, with limiting amount of amoebae. Though unlikely because common (more than 10% of patients), this hypothesis cannot be rejected as a contributing factor for this high number of discordant results. Second, we can speculate that, after positive clinical observation, the practitioner was more likely to conclude about ambiguous amoeba-like cells (e.g. migrating neutrophils). This bias did not seem to be linked to particular practitioners and thus seems to reflect more a trust-worthy biological phenomenon. We can also speculate about the genetic variability in this parasite species, as recently documented [5], and the possible existence of other *Entamoeba* species present in the sulcus. Further studies will permit the assessment of the genetic variability of this parasite and improve diagnostic tools. And finally, as already discussed by Trim et al., the interaction between bacteria and potential amoebic Trojan horses is still to be elucidated in the context of the crevices, since this interplay can lead to the exacerbation of virulence factors of one or both the actors [23].

The prevalence of this disease is still to be agreed on; however, even in countries with a high human development index, periodontitis prevalence reaches 50% in adults above 30 years old [7]. The association of periodontal disease and other pathological conditions – such as diabetes, cardiovascular diseases and pre-term birth – reveals the underestimated importance of this disease for global health [9, 16, 18, 19]. A better, early diagnosis of periodontitis will allow a more accurate determination of individuals at risk for the correlated pathologies. If a causative relationship can be established between periodontitis and these pathologies, therapeutic management of periodontal disease will become part of their prevention, as previously proposed elsewhere [12].

To conclude, this study allowed us to highlight unequivocally that infection by *Entamoeba gingivalis* and periodontitis are correlated. This opens up new perspectives for the understanding and control of this disease, and possibly associated pathologies. Since periodontitis is one of the most prevalent diseases in the world, *E. gingivalis* is a very common parasite among humans. Its identification gives a new target for therapeutic attempts against this disease: anti-parasitic treatments in humans, patient follow-up and experimentation in animal models will allow conclusions about the aetiological link between *E. gingivalis* and periodontitis. The possible significance of targeting the first amoeba discovered in humans for the prevention of other diseases highlights the importance of controlling neglected parasites for public health.

## Online material

Figure S1

Figure S2
