# Supplementary figures and images for: Detection of the amoeba Entamoeba gingivalis in periodontal pockets
Source: Parasite. 2014 Jul 2;21:30. doi: 10.1051/parasite/2014029 (PMC4077299; doi:10.1051/parasite/2014029)

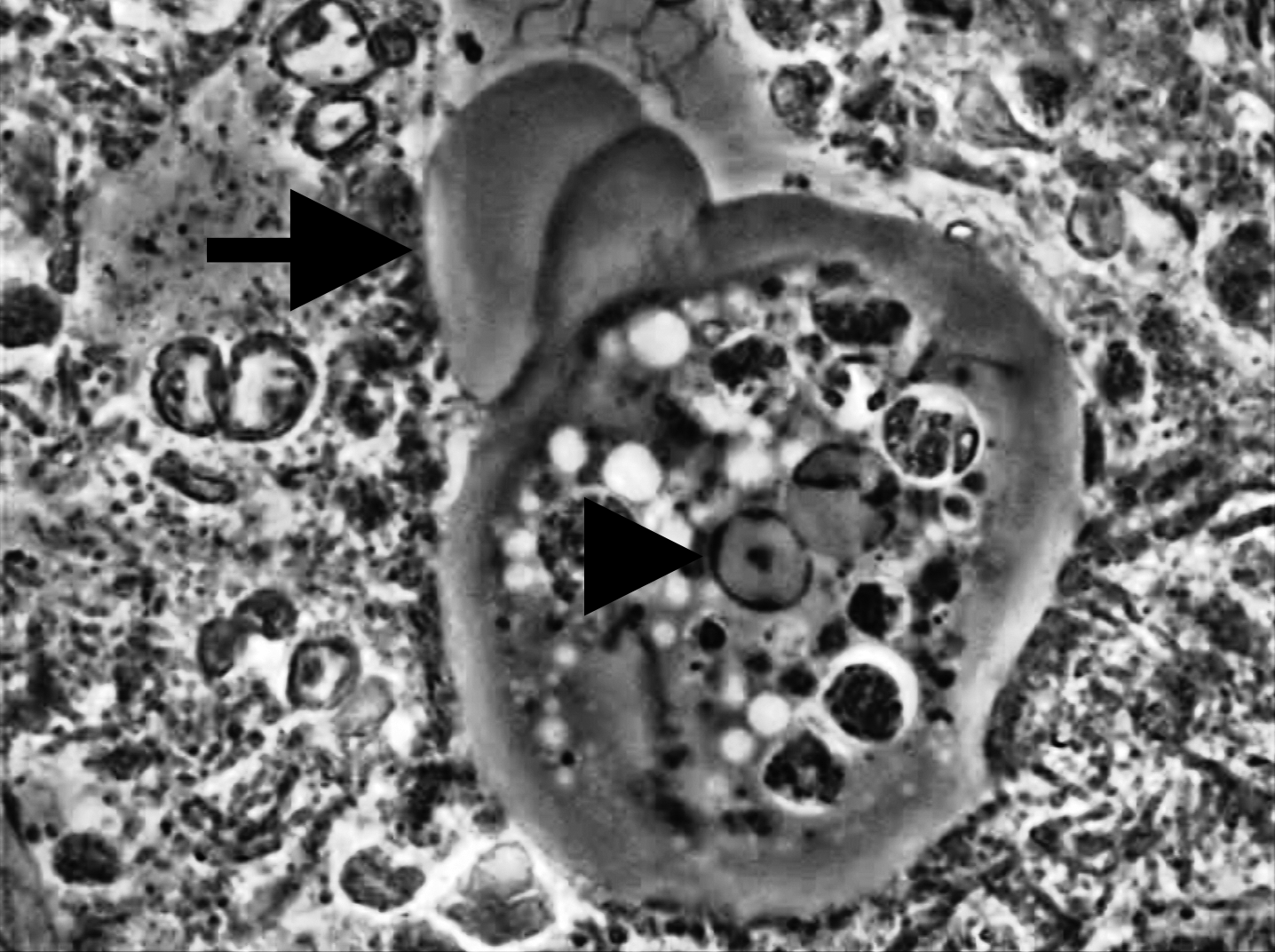

Supplement: Supplementary file 1 — Figure S1 [file parasite-21-30-s1.tif]
