# Supplementary material for: Detection of the amoeba Entamoeba gingivalis in periodontal pockets
Source: Parasite. 2014 Jul 2;21:30. doi: 10.1051/parasite/2014029 (PMC4077299; doi:10.1051/parasite/2014029)
Supplement: Supplementary file 2 — Figure S2 [file parasite-21-30-s2.pdf]

```
Egi   ------------------------ATATGCTGATGTTAAAGATTAAGCCATGCATGTGTA 36
Eco   TATCTGGTTGATCCTGCCAGTATTATATGCTTCTGTCAAAGATTAAGCCATGCATGTCTA 60
Edi   TATCTGGTTGATCCTGCCAGTATTATATGCTGATGTTAGAGATTAAGCCATGCATGTGTA 60
Eha   TATCTGGTTGATCCTGCCAGTATTATATGCTGATGTTAAAGATTAAGCCATGCATGTGTA 60
Ehi   TATCTGGTTGATCCTGCCAGTATTATATGCTGATGTTAAAGATTAAGCCATGCATGTGTA 60
Emo   TATCTGGTTGATCCTGCCAGTATTATATGCTGATGTTAAAGATTAAGCCATGCATGTGTA 60
Epo   TATCTGGTTGATCCTGCCAGTATTATATGCTGATATTAAAGATTAAGCCATGCATGTCTA 60
                              *******  * * * ****************** **

Egi   AGTATAAAGACCAAGAAGGATGAAACTGCGGACGGCTCATTAGAACAGTTATAATTTCTT 96
Eco   AGCACAAAGTCCTAGTATGATGAAGCTGCGAACGGCTCATTACAACAGTTATAATCTTTT 120
Edi   AGTATAAAGACCAAGTAGGATGAAACTGCGGACGGCTCATTATAACAGTAATAGTTTCTT 120
Eha   AGTATAAAGTCCAAGAAGGATGAAACTGCGAACGGCTCATTAGAACAGTTATAGTTTCTT 120
Ehi   AGTATAAAGACCAAGTAGGATGAAACTGCGGACGGCTCATTATAACAGTAATAGTTTCTT 120
Emo   AGTATAAAGACCAAGTAGGATGAAACTGCGGACGGCTCATTATAACAGTAATAGTTTCTT 120
Epo   AGTATAAGTTTTATACAG--CGAAACTGCGGAAGGCTCATTACAACAGTTATAGTTTTGA 118
      ** * **         *    *** ***** * ********* ****** *** * *

Egi   TGATTAGTACCATACAAGGAATAGCT-TTGTGAATAATAAAGATAATACTTGA------- 148
Eco   TGATGAAGTACGTACAAGGA-TATCT-TTGAGAATGTCAAAGCTAATACTTGACGGTTTT 178
Edi   TGGTTAGTAAAGTACAAGGA-TAGCT-TTGTGAATGATAAAGATAATACTTGA------- 171
Eha   CGATTAGTAA-GTACAAGGA-TAGCT-TTGTGAATGATAAAGATAATACTTGA------- 170
Ehi   TGGTTAGTAAAATACAAGGA-TAGCT-TTGTGAATGATAAAGATAATACTTGA------- 171
Emo   TGGTTAGTAAAGTACAAGGA-TAGCT-TTGTGAATGATAAAGATAATACTTGA------- 171
Epo   TGGTTAGTAAAA-AGAAGGA-TAACTCTTGTTAATTGCAGAGCTAATACTTGA------- 169
       * * *       * ***** ** ** ***  ***   * ** **********

Egi   --------GACGA--TC-CTGTTC-TATTACTAGAATAG--------GCGCATT---TCG 185
Eco   CACCCCTTGTCGAGTCCATTCCCTCTGGGAGTGTAATCGATGAGGGCGGGGGATGCTTCA 238
Edi   --------GACGA--TC-CAATTTGTATTAGTACAAAGT--------GGCCAAT---TTA 209
Eha   --------GACGA--TC-TTGGATGTAGAGATACATTCA--------GGCCTTTGCATCA 211
Ehi   --------GACGA--TC-CAGTTTGTATTAGTACAAAAT--------GGCCAAT---TCA 209
Emo   --------GACGA--TC-CGGTTTGTATTAGTACAAGTC--------GGCCACT---CTC 209
Epo   --------TTTTA--CTGTTATCTATATGATTACA------------GCGTAATA----A 203
                  *            *     *  *            *     *

Egi   AACAG--GAATGTAGAAAAGAAGTTTATTAA---GAAAAAGAACAAATT---------TA 231
Eco   CGTATC-CTCTACATATTACCACTTTTTT---TTGAATGAGGGTGGAATATATGCCAAGA 294
Edi   TGTAA--GTAAATTGAGAAATGACATTCTAAGT-GAGTTAGGATGCCAC---------GA 257
Eha   TTTAATGGTGTGAAGAGAAAGGATATCCAAAGT-GAAAGTGGGTATCAT---------GA 261
Ehi   T-TCA--ATGAATTGAGAAATGACATTCTAAGT-GAGTTAGGATGCCAC---------GA 256
Emo   TTCAC--GGGGAGTGCGAA-TGCCATTCTGAATTGAATAAGGATGGTAT---------GA 257
Epo   AGCAATTTATTACATAGGAATAGCTTTTTGA---GAAGAAGGTTAAATT---------GA 251
                        *      *        **    *                  *

Egi   CAATTGTAGAA-ATGAAATACATTTTGACAAGGAATCAATGAAAATATCTGATCTATCAA 290
Eco   GAATTGTAGAAATCGAGAGA--TTTTCACAAGTCATCATTAAGAATATCTGACCTATCAA 352
Edi   CAATTGTAGAACACACAGTG---TTTAACAAGTAACCAATGAGAATTTCTGATCTATCAA 314
Eha   CAATTGTAGAATA-GCGATA---TTTAACAAGTAATCGATGAGAATATCTGATCTATCAA 317
Ehi   CAATTGTAGAACACACAGTG---TTTAACAAGTAACCAATGAGAATTTCTGATCTATCAA 313
Emo   CAATTGTAGAGCACACAGTG---TTTAACAAGTAACCAATGAGAATTTCTGATCTATCAA 314
Epo   GAATTGATGAA-ACTTAATG--TTTTTACAAGTAACTGTTTTAAATATCTGACCTATCAA 308
       *****  **             *** *****  *    *   *** ***** *******

Egi   CT---AGTTGGTAGTATAGAGGACTACCAAGGTTATAACGGATAACGAGAAATTAGGGTT 347
Eco   CTGGTTGTTGGTAAGGTAGTGGCTTACCAAGGTGATAACGGGTAACGAGGAATAAGGGTT 412
Edi   TC---AGTTGGTAGTATCGAGGACTACCAAGATTATAACGGATAACGAGGAATTGGGGTT 371
Eha   CT---AGTTGGTAGTATCGAGGACTACCAAGGTTATAACGGATAACGAGGAATTAGGGTT 374
Ehi   TC---AGTTGGTAGTATCGAGGACTACCAAGATTATAACGGATAACGAGGAATTGGGGTT 370
Emo   TT---TGTTGGTAGTATCGAGGACTACCAAGATTATAACGGATAACGAGGAATTGGGGTT 371
Epo   CT---TGAAGGTATGATAGAGGCATACCTAAGTGATAACGGGTAACGAGAAATAAGGGTT 365
            *  ****   * * **  **** *  * ******* ******* ***  *****

Egi   TGACATCGGAGAAGGAGCTTTCAAAATGGCTACTACTTCTAAGGAAGGCAGCAGGCGCGT 407
Eco   CGACATCGGAGAGGGAGCTTTAGAGATGGCTACCACTTCTAAGGAAGGCAGCAGGCGCGA 472
Edi   CGACATCGGAGAGGGAGCTTTACAGATGGCTACCACTTCTAAGGAAGGCAGCAGGCGCGT 431
Eha   TGACATCGGAGAGGGAGCTTTCAAGATGGCTACCACTTCCAAGGAAGGCAGCAGGCGCGT 434
Ehi   CGACATCGGAGAGGGAGCTTTACAGATGGCTACCACTTCTAAGGAAGGCAGCAGGCGCGT 430
Emo   CGACATCGGAGAGGGAGCTTTACAGATGGCTACCACTTCTACGGAAGGCAGCAGGCGCGT 431
Epo   TGATTTCGGAGAGGGAGCTTTAAAAATGGCTACCACTTCTAAGGAAGGCAGCAGGCGCGC 425
       **  ******* ********  * ******** ***** * *****************
```

```
Egi  AAATTACCCACTTTTAA---CAGAAAGAGGTAGTGACGACAAATAACTCTATTCTTTAAC 464
Eco  AAATTACCCAATCTTAACAAATGAAGGAGGTAGTGACGACAATTAACGCTATCCTCGTCT 532
Edi  AAATTACCCACTTTCGA---ATTGAAGAGGTAGTGACGACACATAACTCTAGAGTTGAGT 488
Eha  AAATTACCCAATTTCAACAGATAGAAGAGGTAGTGACGACAAATAACTCACGACATGCGT 494
Ehi  AAATTACCCACTTTCGA---ATTGAAGAGGTAGTGACGACACATAACTCTAGAGTTGAGT 487
Emo  AAATTACCCACTTTCGA---CGTGAAGAGGTAGTGACGACAAATAACTCTCGAGGTGGTT 488
Epo  AAATTACCCACTTTTAA---TTTAGAGAGGTAGTGACGATAATTAA---TAGAATCGA-T 478
     ********** * *  *         ************* *  ***

Egi  AAA---AAGAATTGAA--GGAATGAACGGAACGTACATAGTTTTGTGAAAGCAATTGGAG 519
Eco  TTTGGCGAGGATCGTC--GGAATGATTTCGATTTAAAATATCGAAAGAAAGCAATTGGAG 590
Edi  AAAATCAATTCTTGAA--GGAATGAGTAGGAGGTAAATTCTCCTACGAAATCAATTGGAG 546
Eha  AAG--CATGTCTTGAA--GGAATGAGTAAGAAGCAAAGAGTCTTACGAAATCAATTGGAG 550
Ehi  AAAATCAATTCTTGAA--GGAATGAGTAGGAGGTAAATTCTCCTACGAAATCAATTGGAG 545
Emo  AACTCCACTTCTTGAA--GGAATGAGTAAGAAGTAAATACTCTTACGAAATCAATTGGAG 546
Epo  ATTTATATTGATTCAAATGTAATGATTAGAGTTTAAAAAAATTTAAGAAATCGATTGGAG 538
                *      * *****         * *         **** * *******

Egi  GGCAAGTCTGGTGCCAGCAGCCGCGGTAATTCCAGCTCCAATAGTATATATTAAAGTTGT 579
Eco  GGCAAGTCTGGTGCCAGCAGCCGCGGTAATTCCAGCTCCAATAGTGTATTGTAAAGTTGT 650
Edi  GGCAAGTCTGGTGCCAGCAGCCGCGGTAATTCCAGCTCCAATAGTGTATATTAAAGTTGC 606
Eha  GGCAAGTCTGGTGCCAGCAGCCGCGGTAATTCCAGCTCCAATAGTGTATATTAAAGTTGT 610
Ehi  GGCAAGTCTGGTGCCAGCAGCCGCGGTAATTCCAGCTCCAATAGTGTATATTAAAGTTGC 605
Emo  GGCAAGTCTGGTGCCAGCAGCCGCGGTAATTCCAGCTCCAATAGTGTATATTAAAGTTGC 606
Epo  GGCAAGTCTGGTGCCAGCAGCCGCGGTAATTCCAGCTCCAATAGTGTATATTAAAGTTGT 598
     ********************************************* ***  ********

Egi  TGTGATTAAAAGGCTCGTAGTTGAATGAAGATA-C-----TATTGA-----AAAGGCTTT 628
Eco  TGTGATTAAAACGCTCGTAGTTGAATGAAAACAATCGGGACAT-GATTTACGATCTCTCC 709
Edi  TGTGATTAAAACGCTCGTAGTTGAATTAAAATG-TGATTTTATACATT-TTGAAGACTT- 663
Eha  TGTGATTAAAACGCTCGTAGTTGAA-AATGATA-TA----TATCCATCGTTCAAGACATG 664
Ehi  TGTGATTAAAACGCTCGTAGTTGAATTAAAATG-TGGTTTTATACATT-TTGAAGACTT- 662
Emo  TGTGATTAAAACGCTCGTAGTTGAATAATAAGG-TGGTTTTAATCATT-TTGAAGGCTT- 663
Epo  TGTGTTTAAAAAGCTCGTAGTCGAAT--------------TAT---------AAGTTTTT 635
     **** ****** ********* ***                *          *

Egi  TCTTT----TTATACAAAAAGAGAAGTTTAG-----TGAA-------ACAATAGAAGA-- 670
Eco  TCCATACGGTGGATTCGTCCATTAGGTCGAGTTGATCAAAA------ATCAAACAATGTC 763
Edi  TANNTA-AGTGAAGTTTCTAGAAATGTTAAA----TTAAA-------ATCAAAGAAGG-- 709
Eha  AGTGTG-ACTTGATCATATGAATGTGTTGAACAACATAAATGGATATAGTAAGGAATG-- 721
Ehi  TATGTA-AGTAAAGTTTCTAGAAATGTTAAA----TTAAA-------ATCAAAGAAGG-- 708
Emo  AGCGCA-AGCTAAGTTTCTAGGAATGAGGTA----ATGAA-------ACTAACGAAGG-- 709
Epo  TATATT-AGTTAA------AAAACTAATATA------AAA---------AAAGGAGAA-- 671
                                           **          *   *

Egi  -------AGG---------A-AATG------------GATTACTTTGAATAAAATAGAGT 701
Eco  CTACTATAAGCGAGAGGGTGGAATTAATTCCAAACTCTTTTACTTTGATTAAATCAAGGT 823
Edi  -------AGACNNTT---CA-AGTA-ATTGAGTTGTTA-TTACTTTGAATAAAATAAGGT 756
Eha  -------ATGCGACA---TG-AATGTGTTGTGTTGTTAATTACTTTGAAAAAATTAGAGT 770
Ehi  -------AAACAATT---CA-AGTA-ATTGAGTTGTTA-TTACTTTGAATAAAATAAGGT 755
Emo  -------AGATGAAG---TG-AGTA-ATCACTTTATCA-TTACTTTGAATAAAATAGAGT 756
Epo  -------AGGTTATT---TATAATC--------TTTTATTTACTTTGAAAAAAATAGAGT 713
            *             * *               *********  ***  *  **

Egi  GTTTAAAGCAAAACA--ATGTTAATGAATAATGAAGCATGGGACA--------AT----- 746
Eco  GCTTAAAGCAAAATCTTCTGTTAACGAATGATGAAGCATGGTATAGAATTAAAACAGAAC 883
Edi  GTTTAAAGCAAAACATTATGTTAATGAATATTCGAGCATGGGACA--------AT---GC 805
Eha  GCTTAAAGCGAA----TCTATTAAAGAATAGTGAAGCATGGGACA--------ATAAAGT 818
Ehi  GTTTAAAGCAAAACATTATGTTAATGAATATTCAAGCATGGGACA--------AT---GC 804
Emo  GTTTAAAGCAAAACATTAAGTTAATGAATATTCAAGCATGGGACA--------AT---GC 805
Epo  GTTTAAAGCAAAA--GTTCATTAATGTATAATGAAGCATAGGATA--------AT----- 758
     * ******* **        **** * **  *  ***** * * *        *

Egi  AAGAAGGA----GATTTGAAAGG-AT---------TTCGAGA------------------ 774
Eco  AGAAAGAATAACGAATAAATGGAGATGGGCAACCATTCGAAATTTATTTATTTTTGTTTT 943
Edi  TGAGGGGA----TGTCAATTAGACAT---------TTCGAGA------------------ 834
Eha  GAATAGGA----GATCTA-CGGATTTCG-----TATTAGGAA------------------ 850
Ehi  TGAGGGGA----TGTCAATAAGACAT---------TTCGAGA------------------ 833
Emo  TGAGGGGA----TGTCT-TCGGACAT---------TTCGAGA------------------ 833
Epo  AATGAGGA----GA------AGAAATT--------TTTGAGA------------------ 782
          * *             *   *         ** *  *
```

```
Egi  -------AGAAGATTAAAAGGAATAATTGGGGTAATTTAGAAAAAGATGGAGAGGTGAA 827
Eco  GTTTAAAACAAGGTTAAGAGAAATGATTAGGGTGATTTAGATAGGAACGGAGAGGTGGA 1003
Edi  -------GAAGGATTAAAAGGAACAATTGGGGTGATTCAGAAAATAACGGGAGAGGTGAA 887
Eha  -------GAAAGATTAAGAGGAATAATTGGGGTAATTCAGAAAATAACGGGAGAGGTGAA 903
Ehi  -------GAAGGATTAAAAGGAACAATTGGGGTGATTCAGAAAATAACGGGAGAGGTGAA 886
Emo  -------GAAGGATTAAAAGGAACAATTGGGGTGATTCAGAAAATAACGGGAGAGGTGAA 886
Epo  -------ACAGTATTAAAAAGGGAAATTGGGGTAATTTAGACAAAATCGGGAGAGGTGAA 835
              *   **** *      *** **** *** *** *     ********** *
Egi  AATCCATGATCATCTTTAGATAAACGAGAGCGAAAGCATTTTACTCA-ATTATATTCATT 886
Eco  ATTCCATGATCGTTTCGAGATAAACGAGAGCGAAAGCATTTCACTGA-ATTCATTTCATT 1062
Edi  AATCCATGATCGGTATAAGATGCACGAGAGCGAAAGCATTTCACTCA-ACTGGGTCCATT 946
Eha  AATTCATGATCGTTATAAGATGAACTAGAGCGAAAGCATTTTACTCA-ATTATGTTCATT 962
Ehi  AATCCATGATCGCTATAAGATGCACGAGAGCGAAAGCATTTCACTCA-ACTGTGTCCATT 945
Emo  ATTCCATGATCGCTATAAGATGCACGAGAGCGAAAGCATTTCACTCA-ACTGGGTCCATT 945
Epo  AATTCATGATCGATTTTGGATAAACTAAAGCGAAAGCATTTTACTCATATTCTTTTATTT 895
     * * *******       ***  ** * ************* *** * * *   *   **
Egi  AATCAAGAACGAAAGTTAGGGGAACAAAGACGATCAGATACCGTCGTAGTCCTAACTATA 946
Eco  AATCAAGAACGAAAGTCAGGGAATCAAAGACGATTAGATACCGTCGTAGTCCTGACCATA 1122
Edi  AATCAAGAACGAAAGTTAGGGGATCGAAGACGATCAGATACCGTCGTAGTCCTAACTATA 1006
Eha  AATCAAGAACGAAAGTTAGGGGATCGAAGACGATCAGATACCGTCGTAGTCCTAACTATA 1022
Ehi  AATCAAGAACGAAAGTTAGGGGATCGAAGACGATCAGATACCGTCGTAGTCCTAACTATA 1005
Emo  AATCAAGAACGAAAGTTAGGGGATCGAAGACGATCAGATACCGTCGTAGTCCTAACTATA 1005
Epo  GATCAAGAACGAAAGTTAGGGGATCGAAGTCGATTAGATACCGTCGTAGTCCTAACTATA 955
      *************** **** * * *** **** *****************  ** ***
Egi  AACGATGTTAACCAAGGATTGGATAAATACATTAATACATTT-TGTATTGTCAT-----T 1000
Eco  AACGATGTCAACTAAGGATTGGATAAG-------AAACATCGAAGTATGAGCCTTTTTCT 1175
Edi  AACGATGTCAACCAAGGATTGGATGAA---ATTCAGATGTACAAAGATGAAGA------- 1056
Eha  AACGATGTCAACGAAGGATTGGATAAA---TTTTAAGACT-----GATAATGATA----T 1070
Ehi  AACGATGTCAACCAAGGATTGGATGAA---ATTCAGATGTACAAAGATAGAGA------- 1055
Emo  AACGATGTCAACCAAGGATTGGATGAA---ATAAAGAAATTCGCGGATGAAGA------- 1055
Epo  AACTATGTCAACTGGGGATTGGATGAA------AATAAAC-----AAGAAAGAT------ 998
     *** **** ***   ********* *        *           *
Egi  GTAAC-TTGTTACAAGAGAATAGAAA---ATGAAAAACAATACTTTATTCAGAACTTGAA 1056
Eco  CCAAC-TTGTTGGAAAAAAAGCGTTTCGCTTCAT--TTCTTCCCTTATTCAGGACTTGAT 1232
Edi  --AACATTGTTTCTAAATCCAAGTAT---ATCAA---TACTACCTTGTTCAGAACTTAAA 1108
Eha  TCAGCATTGCTGGATAACATTTGAAG---TCGAA---TAC-ACCTTATTCAGAACTTGAA 1123
Ehi  --AGCATTGTTTCTAGATCTGAGTAT---ATCAA---TATTACCTTGTTCAGAACTTAAA 1107
Emo  --AACATTGTTTCGGAAACCAAGAGT---TTCAC---AACTACCTTGTTCAGAACTTAAA 1107
Epo  -TAATTTTCATTTTGGAAATT-GATT---TTTATTGTTACTCTCTTATTCAGGATTTAAA 1053
       *   **  *     *     *         *           ** ***** * ** *
Egi  GAGAAATCTT-GAGTGTATGAACTTCAGGGGGAGTATGGTCACAAGGCTGAAACTTAAAG 1115
Eco  GAGAAATCATTAAGTGAATGGACTTCAGGGGGAGTATGGTCACAAGGCTGAAACTTAAAG 1292
Edi  GAGAAATCTT-GAGTTTATGGACTTCAGGGGGAGTATGGTCACAAGGCTGAAACTTAAAG 1167
Eha  GAGAAATCTT-GAGTTATTGGACTTCAGGGGGAGTATGGTCACAAGGCTGAAACTTAAAG 1182
Ehi  GAGAAATCTT-GAGTTTATGGACTTCAGGGGGAGTATGGTCACAAGGCTGAAACTTAAAG 1166
Emo  GAGAAATCTC-GAGTTTATGGACTTCAGGGGGAGTATGGTCACAAGGCTGAAACTTAAAG 1166
Epo  GGGAAACCTTTAAGTGAATGGACTTCAGGGGGAGTATGGTCACAAGGCTGAAACTTAAAG 1113
     * **** *    ***   ** ***************************************
Egi  GAATTGACGGAAGGGCACACCAGGAGTGGAGCCTGCGGCTTAATTTGACTCAACACGGGA 1175
Eco  GAATTGACGGAAGGGCACACCAGGAGTGGAGCCTGCGGCTTAATTTGACTCAACACGGGA 1352
Edi  GAATTGACGGAAGGGCACACCAGGAGTGGAGCCTGCGGCTTAATTTGACTCAACACGGGA 1227
Eha  GAATTGACGGAAGGGCACACAAGGAGTGGAGCCTGCGGCTTAATTTGACTCAACACGGGA 1242
Ehi  GAATTGACGGAAGGGCACACCAGGAGTGGAGCCTGCGGCTTAATTTGACTCAACACGGGA 1226
Emo  GAATTGACGGAAGGGCACACCAGGAGTGGAGCCTGCGGCTTAATTTGACTCAACACGGGA 1226
Epo  GAATTGACGGAAGGGCACACCAGGAGTGGAGCCTGCGGCTTAATTTGACTCAACACGGGA 1173
     ******************** ***************************************
Egi  AAACTTACCAAGACCGAACATAAGAAGGAATGACAGATTAAAGGATCTTTCATGATTGTA 1235
Eco  AAACTTACCAAGACCAAACGGTGTAAGGAATGACAGATTACTGGGTCTTTCATGATTCAT 1412
Edi  AAACTTACCAAGACCGAACAGTAGAAGGAATGACAGATTAAGAGTTCTTTCATGATTTAT 1287
Eha  AAACTTACCAAGACCGAACATTATGAGGAATGACAGATTAAGAGATCTTTCATGATTTAA 1302
Ehi  AAACTTACCAAGACCGAACAGTAGAAGGAATGACAGATTAAGAGTTCTTTCATGATTTAT 1286
Emo  AAACTTACCAAGACCGAACAGTAGAAGGAATGACAGATTAAGAGTTCTTTCATGATTTAT 1286
Epo  AAACTTACCAAGACCGTACATTAAAAGGATTGACAGATTAATAGTTTTTTCATGATATGA 1233
     ***************  **      **** **********   * * *********
```

```
Egi   TGGGGAGTGGTGCATGGCCGTTCTTAGTTGGTGGAATGATTTGTCAGGTTAATTCCGGTA 1295
Eco   CGGGGAGTGGTGCATGGCCGTTCTTAGTTCGTGGACTGATTTGTCTGGTTAATTCCGATA 1472
Edi   TGGGTAGTGGTGCATGGCCGTTCTTAGTTGGTGGAGTGATTTGTCAGGTTAATTCCGGTA 1347
Eha   TGGTGAGTGGTGCATGGCCGTTCTTAGTTGGTGGTATGAATTGTCAGGTTAATTCCGGTA 1362
Ehi   TGGGTAGTGGTGCATGGCCGTTCTTAGTTGGTGGAGTGATTTGTCAGGTTAATTCCGGTA 1346
Emo   TGGGTAGTGGTGCATGGCCGTTCTTAGTTGGTGGAGTGATTTGTCAGGTTAATTCCGGTA 1346
Epo   TGGTGAGTGGTGCATGGCCGTTCTTAGTTGGTGGAGTGATTTGTCTGGTTAATTCCGATA 1293
       **  ************************ ****  *** ***** *********** **
Egi   ACGAACGAGACTTAAACCTATTAATTAGTT-------------GCATTTGAAATGGAAAT 1342
Eco   ACGAACGAGACTTGAACTTATTAATTAGTTGGGCAAGAATCATTTTCGGATGATTTTGCT 1532
Edi   ACGAACGAGACTGAAACCTATTAATTAGTT---------TTCTGCCTATAAGACAGAAAT 1398
Eha   ACGAACGAGACTGAAACCTATTAATTAGTT---------CTGTGCCTATAAGACACAGGT 1413
Ehi   ACGAACGAGACTGAAACCTATTAATTAGTT---------TTCTGCCTATAAGACAGAAAT 1397
Emo   ACGAACGAGACTGAAACCTATTAATTAGTT---------TCCTGCCTATACGACAGGAAT 1397
Epo   ACGAACGAGACTTTATCTTATTAATTGGTT---------T------AGTAAGAGTTTACT 1338
      ************  * * ******** ***                      *      *
Egi   -------GCAGATAATCCTATCATTATTAGAAAT---AATAATACTACTTAAAGGGACAC 1392
Eco   TACCTCCGTAAGGTGGACTGGTGGAATTAAATTT--CGCCTTTACTTCTTAAAGGGACAC 1590
Edi   -------GT---------TCGCAAGAACAGGTGCGTAAGTACCACTTCTTAAAGGGACAC 1442
Eha   -------GT---------TAGCAATAACAGATAG--CGATATCACTTCTTAAAGGGACAC 1455
Ehi   -------GT---------TCGCAAGAACAGGTGCGTAAGTACCACTTCTTAAAGGGACAC 1441
Emo   -------CT---------CCGCAAGGTCAGGTGCCTCGGTACCACTTCTTAAAGGGACAC 1441
Epo   -------TT---------TACTAAAATTATATTT----ATA-TACTATTTAAAAGGACAG 1377
                                  *              ***  ***** *****
Egi   GTATCAATCTCTTTTTTATACAGTAGATAATAATTTGTGTTATTTTTGAATAATAAAAAT 1452
Eco   ATCTTTAATCTTTTCGGGTG---TTCATTG--GTTTCTTTGGGCTTTGC---CCTTAGAT 1642
Edi   ATTTCAATTGTCCTATTTTA------ATTGTTAGTTATCTAATTTC-----GATTAGAAC 1491
Eha   ATTTCAAATGTTC---TTTG------ATTATCAAAGTTGGATTCT-----GTCC--AAC 1499
Ehi   ATTTCAATTGTCCTATTTTA------ATTGT-AGTTATCTAATTTC-----GGTTAGACC 1489
Emo   ATTTCAATTGTCTCATTTTA------ATCG--ATGTCTCTGGCTCC-----GGTCAGAAC 1488
Epo   AC--------TCTCATTTTG-----AATCA--ATTTATTTAATT------------AAAT 1410
                        *       **         *
Egi   TATATAGTGTATCAAAAAGAAAAGATAAAGGAAGCGTTAAGCAATAACAGGTCTGTGATG 1512
Eco   GATATG--AATACCTGAAAAAAAGAAGAAGGAAGCGTCAAGCAATAACAGGTCTGTGATG 1700
Edi   TCTTT----TAACGTGGGAAAAAGAAAAAGGAAGCATTCAGCAATAACAGGTCTGTGATG 1547
Eha   GTGAT----AAACGAAGAAAAAAGAAACAGGAAGCATTCAGCAATAACAGGTCTGTGATG 1555
Ehi   TCTTT----TAACGTGGGAAAAAGAAAAAGGAAGCATTCAGCAATAACAGGTCTGTGATG 1545
Emo   TCTTT----TAACGTGAGAAAAAGAAAAAGGAAGCATTCAGCAATAACAGGTCTGTGATG 1544
Epo   TGATT-------TAAAGAAAGAGGA----GGAAGCATGAAGCAATAACAGGTCTGTGATG 1459
          *              * * **    ****** *  *********************
Egi   CCCTTAGACATCTTGGGCTGCACGCGCGCTACAATGAAAATACTAAAGAGTAGTACCTTA 1572
Eco   CCCTTAGACATCTTGGGCTGCACGCGTGCTACAATGAAAGTACTAACGAGT-CCCCGTTG 1759
Edi   CCCTTAGACATCTTGGGCCGCACGCGCGCTACAATGGAGTTACTAGAGAGCATTTTATCA 1607
Eha   CCCTTAGACATCTTGGGCTGCACGCGCGCTACAATGAAGATAGTAGAGAGTAATATATAT 1615
Ehi   CCCTTAGACATCTTGGGCCGCACGCGCGCTACAATGGAGTTACTAGAGAGTATTTTATCA 1605
Emo   CCCTTAGACATCTTGGGCCGCACGCGCGCTACAATGGAGTTACTAGAGAGTATTTTATCA 1604
Epo   CCCTTAGACATCTTGGGCTGCACGCGTGCTACAATGGAATTTATAGAGAGTAATTTGAAG 1519
      *****************  ******* ********* *  *  **  ***
Egi   CTAGTGGGATTTTTATTCCATTATATTGTATAATGGAGTAA-AAAGAAACAGTAGTAGTA 1631
Eco   TCGGCAATGGGTTGAGTC-------TTTCGGGACGTTGATTCATTGCTGGCAACATTTTC 1812
Edi   TTTACACCTTATTTATTAGGCTATGTC-TAATAGGTAGGG--ATAGTAAGTG--GTGTAC 1662
Eha   ATGACAGTTTCGTAATCATATATTGTT-TACAATGTTGGTT-ATAGAAAGTGTCATAAAC 1673
Ehi   TTTACACCTTATTTATTAGGCTTTGTC-TAATAATTAAGG--ATAGTAAGTG--GTGTAC 1660
Emo   TCCAAGCCTTATATTGTAGACTTTGTT-TATAATGTAGGG--ATATTGGGTG--ATGTAC 1659
Epo   TAAAAGGAAGGTTTAATA-----TATT-TAT-ATATTAGAT-ATGGAATTTTATTTTTAC 1571
                               *      *         *            *
Egi   CAAATTTGAAA-AAAGGAGTAAAACTCA-AAAAATT-------ATACATGACAGGGATTA 1682
Eco   CTAGGCCGAAA-GGCCAAGGAAAGCTCT--AATGTCTTTTTTAATTCTTGACTGGGATGA 1869
Edi   CGAGATTGAAATAGTTAAGGAAAACTCA-AAAGAAC-------GTACATGACAGGGATAA 1714
Eha   CGAAACCGTCA-GGTGGAGGAAAACTTG-AAGCATC-------GTACATGACTGGGATAA 1724
Ehi   CGAGATTGAAATAGTTAAGGAAAACTCA-AAAGAAC-------GTACATGACAGGGATAA 1712
Emo   CTAAATTGAAATAGTTAAGGAAAACTCA-AAAGAAC-------GTACATGACAGGGATAA 1711
Epo   CTATTTTGAAA-AAAGGAGGAAATCTTTTAAAAGGT-------GTACATGACTGGGATTA 1623
      * *    *  *      ** *** **    *             * * **** ***** *
```

```
Egi   ATGATTGAAATTATTTGTTATGAACGAGGAATTCCTTGTAAAATTGTGTCATTAACATAA 1742
Eco   ATGATTGGAATTATTTGTTCTGAACAAGGAATTCCTTGTAAGCGCAAGTCATTACCTTGC 1929
Edi   ATGATTGGAATTATTTGTTTTGAACGAGGAATTCCTTGTAATATCGAGTCATTAACTCGA 1774
Eha   ATGATTGGAATTATTTGTTTTGAACGAGGAATTCCTTGTAATATCACGTCATCAACGTGA 1784
Ehi   ATGATTGGAATTATTTGTTTTGAACGAGGAATTCCTTGTAATATCGAGTCATTAACTCGA 1772
Emo   ATGATTGGAATTATTTGTTTTGAACGAGGAATTCCTTGTAATATCGAGTCATCAACTCGA 1771
Epo   ATGATTGGAATTATTTGTTATGAACGAGGAATTCCTTGTAAGATTTTGTCATTAGCAAAA 1683
      ******* *********** ***** ***************      ***** * *
Egi   GTTGAATACGTCCCTGCCCTTTGTACACACCGCCCGTCGCTCCTACCGATTGAATAAAGA 1802
Eco   GCTGAATAAGTCCCTGCCCTTTGTACACACCGCCCGTCGCTCCTACCGATTGAATAAAGA 1989
Edi   GATGAATACGTCCCTGCCCTTTGTACACACCGCCCGTCGCTCCTACCGATTGAATAAAGA 1834
Eha   GATGAATACGTCCCTGCCCTTTGTACACACCGCCCGTCGCTCCTACCGATTGAATAAAGA 1844
Ehi   GATGAATACGTCCCTGCCCTTTGTACACACCGCCCGTCGCTCCTACCGATTGAATAAAGA 1832
Emo   GATGAATACGTCCCTGCCCTTTGTACACACCGCCCGTCGCTCCTACCGATTGAATAAAGA 1831
Epo   GTTGAATAAGTCCCTGCCCTTTGTACACACCGCCCGTCGCTCCTACCGATTGAATATATA 1743
      * ****** *********************************************** * *
Egi   GGTGAAATTCTAGGA---TTGTTTTTGAGCAATTGAAAATGAAAATAGATGTGAACCTCC 1859
Eco   GGTGAAATGTCAGGA-TTTTGGTGACTTGTCACTGGGAA----AATGATAGTAAATCTCT 2044
Edi   GGTGAAATTCTAGGA-TTCTGTCTTATAG--A-TAGAAA----AATGGATTTAAATCTCC 1886
Eha   GGTGAAATTCTAAGAGTCAAGTAATTCTGTTATTTGACA----AATGGATTTAAATCTCC 1900
Ehi   GGTGAAATTCTAGGA-TTCTGTCTTATAG--A-TAGAAA----AATGGATTTAAATCTCC 1884
Emo   GGTGAAATTCTAGGA-TCTTGCTCTTCGG--AGTGGGAA----AATGGATTTAAATCTCC 1884
Epo   GGTGAAATTTTAGGA-TGATG-AATAGAAATATTTAACA---GAATGGATTTAAATCTCT 1798
      ********   * **     *          * *    *    ***     * ** ***
Egi   TTATTTAGAGGAAGGAGAAGTCGTAACAAGGTTTCCGTAGGTGAACCTGCGGAAGGATC- 1918
Eco   TTGTTTAGAGGAAGGAGAAGTCGTAACAAGGTTTCCGTAGGTGAACCTGCGGAAGGATCA 2104
Edi   TTATTTAGAGGAAGGAGAAGTCGTAACAAGGTTTCCGTAGGTGAACCTGCGGAAGGATCA 1946
Eha   TTATTTAGAGGAAGGAGAAGTCGTAACAAGGTTTCCGTAGGTGAACCTGCGGAAGGATCA 1960
Ehi   TTATTTAGAGGAAGGAGAAGTCGTAACAAGGTTTCCGTAGGTGAACCTGCGGAAGGATCA 1944
Emo   TTATTTAGAGGAAGGAGAAGTCGTAACAAGGTTTCCGTAGGTGAACCTGCGGAAGGATCA 1944
Epo   CTATTTAGAGGAAGGAGAAGTCGTAACAAGGTTTTCGTAGGTGAACCTGCGGAAGGATCA 1858
       * ******************************* ************************
Egi   ------
Eco   ------
Edi   TTA--- 1949
Eha   ------
Ehi   ------
Emo   ------
Epo   ------
```
